# Comparing the Effects of Premedication With Low‐Dose Midazolam, Low‐Dose Etomidate, and Transcutaneous Acupoint Electrical Stimulation on Reducing Etomidate‐Induced Myoclonic Movements and Pain: A Single‐Blind Randomized Clinical Trial

**DOI:** 10.1155/anrp/5199717

**Published:** 2026-11-14

**Authors:** Mehran Rezvani Habibiabadi, Behzad Nazemroaya, Reza Tanhaei

**Affiliations:** ^1^ Department of Anesthesiology and Critical Care School of Medicine Isfahan University of Medical Sciences Isfahan Iran mui.ac.ir

**Keywords:** etomidate, injection pain, midazolam, myoclonus, randomized clinical trial, transcutaneous acupoint electrical stimulation

## Abstract

**Purpose:**

Etomidate is a short‐acting intravenous hypnotic widely used for anesthetic induction because of its favorable hemodynamic profile. However, etomidate frequently causes injection pain and myoclonic movements, which may lead to clinically relevant complications in susceptible patients. This randomized clinical trial evaluated whether premedication with low‐dose midazolam, low‐dose etomidate, or transcutaneous acupoint electrical stimulation (TAES) reduces etomidate‐induced myoclonus and injection pain compared with control in adults undergoing elective surgery.

**Methods:**

In this single‐blind (assessor‐blinded), randomized, parallel‐group clinical trial, 92 adult patients (18–60 years, ASA physical Status I–II) scheduled for elective surgical procedures at Kashani Hospital (Isfahan, Iran) were allocated to four groups (*n* = 23 each): control (normal saline), midazolam (0.15 mg/kg), etomidate (0.03 mg/kg), and TAES. TAES was applied bilaterally to the Hegu (LI4), Neiguan (PC6), and Zusanli (ST36) acupoints 30 min before etomidate injection. Injection pain was assessed using the Ambesh Four‐Point Behavioral Scale. The presence, severity (0–3), and duration (< 5 s, 5–10 s, > 10 s) of etomidate‐induced myoclonic movements were recorded. Hemodynamic parameters were monitored at predefined time points. The primary outcome was the combined incidence of injection pain and myoclonic movements during etomidate administration. Secondary outcomes included myoclonus severity and duration, individual pain and myoclonus scores, periprocedural hemodynamics, and adverse events. Statistical analysis used chi‐square or Fisher’s exact tests and one‐way ANOVA, with *p* < 0.05 considered significant.

**Results:**

Ninety‐two patients completed the trial (23 per group). Age, height, weight, and baseline hemodynamic parameters were comparable among groups, whereas sex distribution and the prevalence of some comorbidities differed significantly. The incidence of injection pain differed significantly among groups (*p* = 0.006): 17.4% (*n* = 4) of patients in the control group exhibited behavioral signs of pain, whereas no injection pain was observed in the midazolam, etomidate, or TAES groups. Myoclonic movements were observed in 60.9% (*n* = 14) of patients in the control group, compared with 26.1% (*n* = 6), 39.1% (*n* = 9), and 34.8% (*n* = 8) in the etomidate, midazolam, and TAES groups, respectively (*p* = 0.099). The incidence of the composite primary outcome (defined as injection pain and/or myoclonus) was 60.9% in the control group, compared with 26.1%, 39.1%, and 34.8% in the etomidate, midazolam, and TAES groups, respectively. Compared with control, the risk of the composite endpoint was reduced in all intervention groups (etomidate RR 0.43, 95% CI 0.20–0.92; midazolam RR 0.64, 95% CI 0.35–1.18; TAES RR 0.57, 95% CI 0.30–1.09). The distribution of myoclonus severity (*p* = 0.180) and duration (*p* = 0.180) did not differ significantly between groups. No clinically relevant hemodynamic instability, respiratory depression, or major complications were observed.

**Conclusion:**

Premedication with low‐dose midazolam, low‐dose etomidate, or TAES completely prevented observable injection pain and produced a clinically meaningful, though statistically nonsignificant, reduction in the incidence of etomidate‐induced myoclonic movements compared with control, without compromising hemodynamic stability. These interventions may represent safe and practical strategies to mitigate common adverse effects associated with etomidate; however, larger multicenter trials are warranted to confirm their efficacy for preventing myoclonus.

**Trial Registration:** Iranian Registry of Clinical Trials: IRCT20160307026950N70

## 1. Introduction

Etomidate is a short‐acting intravenous anesthetic agent widely used for induction of anesthesia because of its rapid onset, favorable pharmacokinetic profile, and relative hemodynamic stability [1, 2]. Despite these advantages, etomidate is associated with several adverse effects, including injection pain, myoclonic movements, nausea, vomiting, and adrenal suppression [3, 4]. Myoclonus, occurring in up to 80% of patients, is one of the most frequent and troublesome side effects.

Although etomidate‐induced myoclonus is generally self‐limited and not considered a disease entity per se [5], it may have important clinical consequences. In vulnerable patients, myoclonus can increase intraocular pressure, intracranial pressure, or cardiovascular stress and may precipitate regurgitation and aspiration or exacerbate underlying cardiovascular disease [16, 7]. Consequently, strategies to prevent or attenuate this adverse effect are clinically relevant, particularly in high‐risk populations.

An ideal prophylactic intervention to prevent etomidate‐induced myoclonus should not interfere with the desirable pharmacodynamic characteristics of etomidate or cause significant adverse effects. A variety of pharmacologic agents have been investigated, including benzodiazepines, opioids, and muscle relaxants [8‐12]. Benzodiazepines such as midazolam can attenuate myoclonic movements through γ‐aminobutyric acid (GABA)–mediated inhibitory mechanisms, but their use may be limited by excessive sedation or respiratory depression in some patients [13]. Opioids, including μ‐ and κ‐receptor agonists, and neuromuscular blocking agents have also shown varying degrees of effectiveness; however, concerns about respiratory depression, chest wall rigidity, or interference with neurologic monitoring may restrict their routine use.

Low‐dose etomidate itself has been proposed as a strategy to reduce myoclonus, based on the concept of receptor desensitization. Pretreatment with a small dose of etomidate may partially saturate cortical and subcortical receptors, thereby decreasing the excitatory motor manifestations associated with the subsequent full induction dose. Some clinical studies have reported significantly lower incidence and severity of myoclonus in patients pretreated with low‐dose etomidate compared with other agents such as magnesium sulfate, midazolam, or remifentanil [6].

Nonpharmacologic approaches have also emerged as potential alternatives or adjuncts. Acupoint electrical stimulation (AES), rooted in Traditional Chinese Medicine, has been evaluated for perioperative analgesia and modulation of intraoperative anesthetic requirements. Transcutaneous acupoint electrical stimulation (TAES) is a non‐invasive technique derived from acupuncture, in which surface electrodes are placed on specific acupoints and stimulated electrically. TAES has been shown to exert analgesic effects, enhance anesthetic efficacy, and reduce the required doses of anesthetic drugs [14]. Proposed mechanisms involve activation of endogenous opioid pathways and increased secretion of β‐endorphin, enkephalin, and dynorphin, which can produce effects similar to opioid drugs [15].

Clinical studies have suggested that TAES, particularly when combined with low‐dose opioids such as sufentanil, may reduce the incidence and severity of etomidate‐induced myoclonus. In one trial, TAES plus low‐dose sufentanil significantly decreased myoclonic movements compared with control [16]. Other investigations have compared various pharmacologic pretreatments, including midazolam, magnesium sulfate, remifentanil, and low‐dose etomidate, with mixed results regarding their relative efficacy and safety profiles [17].

Despite these efforts, there is no clear consensus on the optimal strategy to prevent etomidate‐induced myoclonus and injection pain, and comparative data involving both pharmacologic and nonpharmacologic modalities remain limited, particularly in the context of domestic practice.

Therefore, this randomized clinical trial was designed to compare the effects of premedication with low‐dose midazolam, low‐dose etomidate, and TAES on etomidate‐induced myoclonic movements and injection pain, as well as on hemodynamic stability, in adult patients undergoing elective surgical procedures. We hypothesized that each of these interventions would reduce the incidence of myoclonus and injection pain compared with control, without causing clinically significant hemodynamic compromise.

## 2. Materials and Methods

### 2.1. Study Design and Setting

This single‐blind (assessor‐blinded), randomized, parallel‐group clinical trial was conducted from 2024 to 2025 at Kashani Hospital, affiliated with Isfahan University of Medical Sciences (Isfahan, Iran). The study protocol was reviewed and approved by the Ethics Committee for Research at Isfahan University of Medical Sciences (approval number IR.MUI.MED.REC.1404.070) and complied with the principles of the Declaration of Helsinki (revised 2013).

### 2.2. Participants

Eligible participants were adult patients aged 18–60 years with American Society of Anesthesiologists (ASA) physical status I or II, scheduled for elective surgical procedures for which anesthetic induction with etomidate was planned.

Exclusion criteria were BMI < 18 or > 31 kg/m^2^; ASA physical status III or higher; history of heart failure or heart block; hepatic or renal disease; confusion or dementia; known allergy to any study drug; severe neurologic or psychiatric disorders; pregnancy or breastfeeding; use of analgesic drugs within 24 h before surgery; emergency surgery; thrombophlebitis; history of seizures; chronic pain; long‐term use of sedatives or analgesics; and any change in anesthetic technique during the study period.

All participants provided written informed consent before enrollment.

### 2.3. Sample Size

The required sample size was calculated for four independent groups, assuming a Type I error of 5% (*α* = 0.05), statistical power of 80% (1 − *β* = 0.80), and an effect size of 1 (Δ/*σ* = 1) based on expected differences in the incidence of myoclonus among groups. According to standard sample size tables for one‐way ANOVA, a minimum of 23 patients per group was required. Thus, 92 patients (4 × 23) were enrolled.

### 2.4. Randomization and Blinding

Patients were recruited using convenience sampling and subsequently randomized to one of four study groups (control, midazolam, etomidate, and TAES) using a computer‐generated random sequence. Group allocation was concealed in sequentially numbered, opaque, sealed envelopes that were opened immediately before the intervention.

The study was single‐blind in that the outcome assessor and data analyst were blinded to group allocation. Participant blinding was not feasible due to the visible nature of the TAES intervention (placement of electrodes and stimulation). The anesthesiologist responsible for applying TAES and administering premedication was not involved in outcome assessment or data analysis.

### 2.5. Study Groups and Interventions

Patients were allocated to one of the following four groups (*n* = 23 in each):

• Control (C): 2 mL normal saline (intravenous).
• Midazolam (M): 0.15 mg/kg midazolam (intravenous).
• Etomidate (E): 0.03 mg/kg etomidate (intravenous).
• TAES (S): TAES applied before etomidate injection.

Premedication in the pharmacologic groups (control, midazolam, and etomidate) was administered via a 20‐G intravenous catheter inserted into a vein on the dorsum of the hand. In the TAES group, no intravenous premedication was given; instead, TAES was applied as described below.

### 2.6. TAES Protocol

In the TAES group, TAES was initiated 30 min before etomidate injection using a portable acupoint nerve stimulator (model KWD‐808, Changzhou Yingdi Electronic Co., China). Three pairs of adhesive surface electrodes were placed bilaterally at the Hegu (LI4), Neiguan (PC6), and Zusanli (ST36) acupoints, identified according to standard anatomical landmarks (Figure 1). Hegu (LI4) is located on the dorsum of the hand between the first and second metacarpal bones, Neiguan (PC6) on the volar aspect of the forearm approximately 2–3 cm proximal to the wrist crease between the tendons of palmaris longus and flexor carpi radialis, and Zusanli (ST36) about 3–4 cm below the lateral border of the tibial tuberosity. Electrical stimulation was delivered in a dense–disperse mode with alternating frequencies of 2 Hz and 100 Hz. The current intensity was gradually increased until patients reported a mild but tolerable tingling sensation without visible muscle contraction (typically 6–10 mA). Each TAES session lasted 30 min and was performed by an anesthesiologist trained in acupuncture‐related procedures. The skin at each acupoint was cleaned with 70% alcohol prior to electrode placement to ensure safety and optimal conductivity.

**FIGURE 1:**
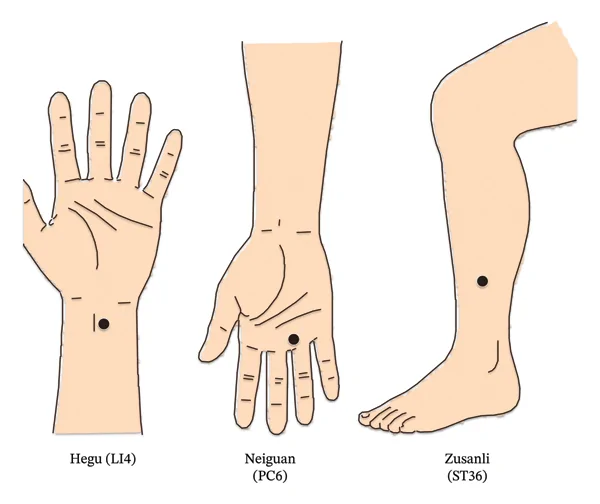
Anatomical locations of the three acupoints used for TAES: Hegu (LI4), Neiguan (PC6), and Zusanli (ST36).

### 2.7. Outcome Measures

The primary outcome was the combined incidence of etomidate‐induced myoclonic movements and injection pain during etomidate administration, defined as the occurrence of either behavioral signs of injection pain, myoclonic movements, or both.

Secondary outcomes included individual incidence of myoclonic movements; myoclonus severity grade (0–3); myoclonus duration (< 5 s, 5–10 s, > 10 s); injection pain grade (Ambesh Four‐Point Behavioral Scale); periprocedural hemodynamic parameters (heart rate [HR], systolic blood pressure [SBP], diastolic blood pressure [DBP], mean arterial pressure [MAP], and peripheral oxygen saturation [SpO_2_]) before and after etomidate injection; and adverse events such as respiratory depression, nausea, vomiting, or other complications.

### 2.8. Assessment of Injection Pain

Injection pain was evaluated using the Ambesh Four‐Point Behavioral Pain Scale, which has been widely applied in studies assessing injection pain associated with etomidate and propofol. The scale is based on observable behavioral responses and is scored as follows:

 0 = *no pain*.
 1 = *mild pain* (facial grimacing or slight movement).
 2 = *moderate pain* (withdrawal of the hand or pronounced movement).
 3 = *severe pain* (strong vocalization or vigorous withdrawal).

Pain assessment was performed during the first 5 s after the start of etomidate injection, before loss of consciousness and before the onset of any involuntary muscle activity. Because patients typically lost consciousness within seconds under etomidate, self‐reported scales such as the Visual Analog Scale (VAS) or Numeric Rating Scale (NRS) were not feasible; therefore, behavioral assessment was considered the most reliable and objective method.

### 2.9. Assessment of Myoclonic Movements

Myoclonic movements were assessed immediately after completion of the etomidate injection and before full induction of anesthesia. To distinguish clearly between injection pain and subsequent myoclonic activity, the temporal sequence of assessment was strictly maintained: pain scoring during the first 5 s of injection, followed by observation for myoclonus.

The severity of myoclonus was graded from 0 to 3:

 0 = no movement.
 1 = mild (movement limited to the wrist or one limb).
 2 = moderate (involving two or more limbs or trunk).
 3 = severe (generalized movements involving multiple limbs or associated with apnea, nausea, or vomiting).

The duration of myoclonic movements was categorized as

 < 5 s.
 5–10 s.
 > 10 s.

These assessments allowed separate evaluation of the presence, severity, and duration of etomidate‐induced myoclonus.

### 2.10. Hemodynamic Monitoring

Hemodynamic parameters, including HR, SBP, DBP, and MAP, were monitored to evaluate hemodynamic stability throughout the observation period. Measurements were obtained at three predefined time points:

1. Baseline: immediately before administration of the study intervention (premedication or initiation of TAES).
2. Preinjection: immediately before etomidate administration.
3. Postinjection: 1 minute after completion of etomidate injection.

All measurements were performed using a standard noninvasive multiparameter monitor (Datex‐Ohmeda, Helsinki, Finland) integrated into the anesthesia workstation. The mean of two consecutive readings at each time point was used for analysis to minimize random measurement variability. Continuous electrocardiographic monitoring and pulse oximetry were maintained for all patients.

### 2.11. Statistical Analysis

Statistical analysis was performed using SPSS software (Version 28, IBM Corp., Armonk, NY, USA). Qualitative variables were expressed as frequencies and percentages and compared among groups using the chi‐square test or Fisher’s exact test, as appropriate. Quantitative variables were tested for normality using the Shapiro–Wilk test and expressed as mean ± standard deviation (SD). Intergroup comparisons of normally distributed data were performed using one‐way analysis of variance (ANOVA). A two‐sided *p* value < 0.05 was considered statistically significant.

To address potential baseline imbalances identified after randomization, additional sensitivity analyses were conducted using multivariable regression models adjusting for sex, hypertension, and epilepsy as covariates.

Following significant omnibus testing, Bonferroni‐adjusted post hoc pairwise comparisons were performed to localize between‐group differences in injection pain.

In addition to hypothesis testing, effect sizes with 95% confidence intervals (CIs) were calculated for binary outcomes (risk ratios and absolute risk differences). Analyses were performed on an intention‐to‐treat basis (all randomized participants analyzed in their allocated group). For outcomes with zero events in one or more groups, an appropriate continuity correction was applied when estimating relative effects.

Sensitivity analyses adjusting for sex distribution, hypertension, and epilepsy yielded effect estimates consistent with the primary analysis, indicating that baseline imbalance did not materially alter the direction or magnitude of treatment effects.

## 3. Results

### 3.1. Patient Characteristics

A total of 92 patients were enrolled and randomized equally into the four groups (*n* = 23 per group). All participants completed the study, with no losses to follow‐up or exclusions after randomization. The flow of participants through the study is illustrated in Figure 2.

**FIGURE 2:**
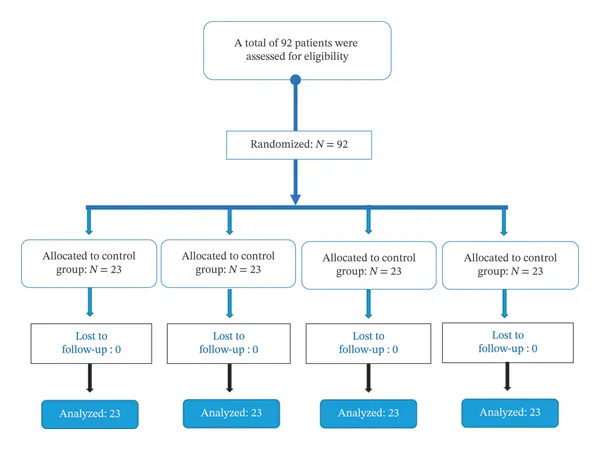
CONSORT flow diagram of participant enrollment, allocation, follow‐up, and analysis.

*Participant flow*: Of 92 randomized participants, 92/92 received the allocated intervention (23 per group) and were included in the primary analysis; there were no losses to follow‐up, withdrawals, or exclusions after randomization.

Overall, 63% (*n* = 58) of participants were female and 37% (*n* = 34) were male. The proportion of female patients differed significantly among groups: 43.5% (*n* = 10) in the control group, 47.8% (*n* = 11) in the etomidate group, 82.6% (*n* = 19) in the midazolam group, and 78.3% (*n* = 18) in the TAES group. Correspondingly, male representation was 56.5% (*n* = 13), 52.2% (*n* = 12), 17.4% (*n* = 4), and 21.7% (*n* = 5) in the control, etomidate, midazolam, and TAES groups, respectively (*p* = 0.007) (Table 1).

**TABLE 1:** Distribution of demographic characteristics and medical history across the study groups.

Variable	Control (*n* = 23)	Etomidate (*n* = 23)	Midazolam (*n* = 23)	TAES (*n* = 23)	*p* value
Gender					0.007
Female	10 (43.5%)	11 (47.8%)	19 (82.6%)	18 (78.3%)	
Male	13 (56.5%)	12 (52.2%)	4 (17.4%)	5 (21.7%)	
Medical history					
Asthma	4 (17.4%)	2 (8.7%)	7 (30.4%)	7 (30.4%)	0.2573
Diabetes	4 (17.4%)	2 (8.7%)	3 (13.0%)	1 (4.3%)	0.5489
Epilepsy	5 (21.7%)	1 (4.3%)	8 (34.8%)	10 (43.5%)	0.0318
Hypertension	5 (21.7%)	13 (56.5%)	4 (17.4%)	5 (21.7%)	0.0273
No disease	5 (21.7%)	5 (21.7%)	1 (4.3%)	0 (0.0%)	0.0455

*Note:* Values are presented as frequency (percentage).

Regarding comorbidities, 21.7% (*n* = 20) of patients had a history of asthma, 10.9% (*n* = 10) diabetes, 26.1% (*n* = 24) epilepsy, 29.3% (*n* = 27) hypertension, and 12.0% (*n* = 11) had no known diseases. The distribution of these medical histories differed significantly among groups based on the Phi test (*p* = 0.004), with statistically significant group differences particularly for epilepsy, hypertension, and absence of comorbid disease.

Baseline demographic and clinical characteristics of the study population are presented in Table 1.

In contrast, age, height, weight, and baseline hemodynamic parameters were comparable among groups. Baseline hemodynamic parameters are summarized in Table 2.

**TABLE 2:** Comparison of mean age, height, weight, blood pressure, and heart rate among study groups.

Variable	Control (*n* = 23)	Etomidate (*n* = 23)	Midazolam (*n* = 23)	TAES (*n* = 23)	*p* value
Age (years)	41.4 ± 16.7	47.3 ± 16.0	47.4 ± 18.1	51.5 ± 14.3	0.384
Height (cm)	165.7 ± 9.1	164.0 ± 15.3	171.2 ± 12.5	169.3 ± 9.5	0.266
Weight (kg)	73.7 ± 8.9	74.0 ± 8.7	76.7 ± 12.2	73.8 ± 9.6	0.876
Systolic BP (mmHg)	117.0 ± 11.0	118.0 ± 11.0	122.3 ± 12.0	121.9 ± 9.7	0.398
Diastolic BP (mmHg)	73.8 ± 9.3	77.6 ± 18.6	75.7 ± 10.6	74.7 ± 11.3	0.956
Heart rate (bpm)	75.4 ± 18.8	72.1 ± 10.5	72.4 ± 6.0	74.9 ± 9.8	0.648

*Note:* Values are presented as mean ± standard deviation.

Mean ages were 41.4 ± 16.7, 47.3 ± 16.0, 47.4 ± 18.1, and 51.5 ± 14.3 years in the control, etomidate, midazolam, and TAES groups, respectively (*p* = 0.384). Mean body weights were 73.7 ± 8.9, 74.0 ± 8.7, 76.7 ± 12.2, and 73.8 ± 9.6 kg (*p* = 0.876), and mean heights were 165.7 ± 9.1, 164.0 ± 15.3, 171.2 ± 12.5, and 169.3 ± 9.5 cm (*p* = 0.266), respectively. Baseline HR and blood pressure values did not differ significantly among groups.

### 3.2. Hemodynamic Parameters

The mean baseline HRs before etomidate injection were 75.4 ± 18.8, 72.1 ± 10.5, 72.4 ± 6.0, and 74.9 ± 9.8 beats/min in the control, etomidate, midazolam, and TAES groups, respectively (*p* = 0.648). Systolic and DBPs showed no significant differences among groups before or after etomidate injection (*p* = 0.398 and *p* = 0.956, respectively) (Table 2). Throughout the observation period, no clinically meaningful hemodynamic instability was observed in any group.

### 3.3. Injection Pain

To account for baseline imbalances in sex distribution and selected comorbidities (epilepsy and hypertension), multivariable logistic regression analysis was performed. After adjustment, each intervention remained associated with significantly lower odds of injection pain compared with control (low‐dose etomidate OR 0.18, 95% CI 0.04–0.79; midazolam OR 0.22, 95% CI 0.05–0.91; TAES OR 0.20, 95% CI 0.04–0.87), confirming robustness of the primary findings.

Post hoc Bonferroni‐adjusted pairwise comparisons demonstrated significant differences between each intervention group and the control group, while no statistically significant differences were observed among the intervention groups.

Table 3 has been expanded to include explicit injection pain incidence data to improve transparency and interpretability.

**TABLE 3:** Incidence and severity distribution of injection pain.

Variable	Control (*n* = 23)	Etomidate (*n* = 23)	Midazolam (*n* = 23)	TAES (*n* = 23)	*p* value
Pain					0.006
Absence	19 (82.6%)	23 (100%)	23 (100%)	23 (100%)	
Present	4 (17.4%)	0 (0%)	0 (0%)	0 (0%)	
1 (*mild*)	2 (8.7%)	0	0	0	
2 (*moderate*)	1 (4.3%)	0	0	0	
3 (*severe*)	1 (4.3%)	0	0	0	

*Note:* Injection pain was assessed within the first 5 s after etomidate injection using the Ambesh Four‐Point Behavioral Scale (0–3). Data are presented as numbers (percentage). Intergroup comparisons were performed using Fisher’s exact test with continuity correction due to zero‐event cells.

Abbreviation: TAES, transcutaneous acupoint electrical stimulation.

The group difference in the incidence of injection pain was statistically significant (*p* = 0.006) (Table 3). These findings indicate that all three interventions—low‐dose midazolam, low‐dose etomidate, and TAES—were highly effective in preventing observable injection pain associated with etomidate.

Effect size: Compared with control, injection pain was lower in each intervention group (absolute risk difference −17.4%, 95% CI −32.9% to −1.9%).

Analysis of the composite endpoint demonstrated that the observed between‐group differences were primarily driven by reductions in injection pain, whereas the contribution of myoclonus incidence was modest and did not reach statistical significance. Co‐occurrence of injection pain and myoclonus was observed only in the control group.

Detailed distributions of injection pain incidence and severity are presented in Table 4 to enhance transparency and interpretability of group comparisons.

**TABLE 4:** Comparison of frequency distribution of presence, severity, and duration of myoclonus movements.

Variable	Control (*n* = 23)	Etomidate (*n* = 23)	Midazolam (*n* = 23)	TAES (*n* = 23)	*p* value
Myoclonus presence					0.099
Absence	9 (39.1%)	17 (73.9%)	14 (60.9%)	15 (65.2%)	
Present	14 (60.9%)	6 (26.1%)	9 (39.1%)	8 (34.8%)	
Severity					0.180
0 (none)	9 (39.1%)	17 (73.9%)	14 (60.9%)	15 (65.2%)	
1 (mild)	5 (21.7%)	3 (13.0%)	5 (21.7%)	5 (21.7%)	
2 (moderate)	9 (39.1%)	3 (13.0%)	4 (17.4%)	2 (8.7%)	
3 (severe)	0 (0.0%)	0 (0.0%)	0 (0.0%)	1 (4.3%)	
Duration					0.180
< 5 s	5 (21.7%)	3 (13.0%)	5 (21.7%)	5 (21.7%)	
5–10 s	9 (39.1%)	3 (13.0%)	4 (17.4%)	2 (8.7%)	
> 10 s	0 (0.0%)	0 (0.0%)	0 (0.0%)	1 (4.3%)	

*Note:* Severity scores: 0 = no movement, 1 = mild, 2 = moderate, 3 = severe. Duration categories: < 5 s, 5–10 s, > 10 s.

The incidence of the composite primary endpoint—defined as the occurrence of injection pain, myoclonus, or both—was 60.9% (14/23) in the control group, compared with 26.1% (6/23), 39.1% (9/23), and 34.8% (8/23) in the etomidate, midazolam, and TAES groups, respectively. Compared with control, the risk of the composite outcome was lower in all intervention groups: etomidate (RR 0.43, 95% CI 0.20–0.92), midazolam (RR 0.64, 95% CI 0.35–1.18), and TAES (RR 0.57, 95% CI 0.30–1.09). These findings indicate a clinically meaningful reduction in the composite endpoint, although statistical significance was not reached across all comparisons.

Due to zero‐event rates for injection pain in the intervention groups, the composite outcome was primarily driven by the incidence of myoclonic movements, while co‐occurrence of pain and myoclonus was observed only in the control group. The incidence and severity distribution of injection pain are presented in Table 4.

The incidence of the composite primary outcome is presented in Table 5.

**TABLE 5:** Incidence of the composite primary outcome (injection pain and/or myoclonus).

Group	Composite outcome (*n*, %)	Risk ratio (95% CI) vs. control
Control	14 (60.9%)	Reference
Etomidate	6 (26.1%)	0.43 (0.20–0.92)
Midazolam	9 (39.1%)	0.64 (0.35–1.18)
TAES	8 (34.8%)	0.57 (0.30–1.09)

*Note:* The composite outcome was defined as the occurrence of injection pain, myoclonus, or both. Due to zero‐event rates for injection pain in the intervention groups, the composite endpoint was primarily driven by the incidence of myoclonus. Risk ratios were calculated using standard methods, and confidence intervals are reported at the 95% level.

### 3.4. Myoclonic Movements

The incidence, severity, and duration of myoclonic movements are summarized in Table 3. A comparison of etomidate‐induced myoclonic movements demonstrated that 60.9% (*n* = 14) of patients in the control group, 26.1% (*n* = 6) in the etomidate group, 39.1% (*n* = 9) in the midazolam group, and 34.8% (*n* = 8) in the TAES group exhibited myoclonus. Although this distribution suggested a clinically relevant reduction in myoclonus incidence among all intervention groups compared with control, the intergroup difference did not reach statistical significance (*p* = 0.099) (Table 3).

Effect size (any myoclonus vs. control): low‐dose etomidate RR 0.43 (95% CI 0.20–0.92); midazolam RR 0.64 (95% CI 0.35–1.18); TAES RR 0.57 (95% CI 0.30–1.09).

Regarding severity, mild myoclonic movements (Grade 1) occurred in 21.7% (*n* = 5), 13.0% (*n* = 3), 21.7% (*n* = 5), and 21.7% (*n* = 5) of patients in the control, etomidate, midazolam, and TAES groups, respectively. Moderate myoclonus (Grade 2) occurred in 39.1% (*n* = 9), 13.0% (*n* = 3), 17.4% (*n* = 4), and 8.7% (*n* = 2), respectively. Severe myoclonus (Grade 3) was rare and observed in only one patient (4.3%) in the TAES group (*p* = 0.180).

The duration of myoclonic movements was < 5 s in 21.7% of patients, 5–10 s in 39.1%, and > 10 s in only one case in the TAES group, with no significant differences in duration distribution among groups (*p* = 0.180) (Table 3).

### 3.5. Adverse Events

No patient in any group experienced respiratory depression, significant desaturation, clinically relevant arrhythmia, or any other major complication during the observation period. All 92 patients completed the study protocol, and their data were included in the final analysis.

## 4. Discussion

This study assessed whether premedication with low‐dose midazolam, low‐dose etomidate, or TAES reduces etomidate‐induced myoclonus and injection pain. A statistically significant difference in the incidence of observable injection pain was identified among the study groups (*p* = 0.006). Observable injection pain was reported in 17.4% of patients in the control group, while no such events occurred in the midazolam, etomidate, or TAES groups. These results indicate that all three interventions are effective in preventing observable etomidate injection pain.

Each intervention group demonstrated a lower incidence of myoclonic movements compared to the control group. Myoclonus was observed in 60.9% of patients in the control group, 26.1% in the etomidate group, 39.1% in the midazolam group, and 34.8% in the TAES group. However, the overall difference between groups was not statistically significant (*p* = 0.099). No significant differences were found in myoclonus severity (*p* = 0.180) or duration (*p* = 0.180) among the groups. Therefore, the reductions in myoclonus should be regarded as a favorable clinical trend rather than conclusive evidence of efficacy.

These results align with previous research. Dey and Kumar reported a significantly lower incidence of myoclonus with dexmedetomidine compared to midazolam, underscoring the potential of pre‐induction sedatives to attenuate etomidate‐related excitatory activity [1]. Similarly, Alipour et al. found sufentanil to be more effective than midazolam in preventing myoclonic movements [2]. Lv et al. demonstrated that TAES combined with low‐dose opioids, such as sufentanil, reduced both the incidence and severity of etomidate‐induced myoclonus [3]. Furthermore, Sedighinejad et al. observed significantly lower incidence and severity of myoclonus in patients receiving low‐dose etomidate compared to those receiving magnesium sulfate, midazolam, or remifentanil [4]. Salim et al. also reported a preventive effect of midazolam, with abnormal muscle movements absent in 84.5% of patients who received midazolam pretreatment [5]. Collectively, these studies support the reductions observed with low‐dose etomidate and midazolam in the present trial. However, the between‐group comparison of myoclonus incidence in this study was not statistically significant.

The reduction in injection pain observed with TAES in this study is consistent with findings by Feng et al., who reported that TAES may enhance perioperative analgesia and reduce anesthetic requirements [6]. One proposed mechanism involves activation of endogenous opioid pathways and increased release of β‐endorphin, enkephalin, and dynorphin, as described by Lee et al. [7]. These mechanisms may partially account for the absence of observable injection pain and the lower incidence of myoclonic movements in the TAES group.

Hemodynamic parameters, including HR and blood pressure, remained stable before and after etomidate injection in all study groups. This observation is consistent with Hüter et al., who reported that co‐administration of midazolam and etomidate maintained hemodynamic stability and reduced excitatory movements during induction [8]. Additionally, a network meta‐analysis by Zhang et al. identified pretreatment with low‐dose etomidate and selected opioid receptor agonists as strategies associated with reductions in moderate‐to‐severe etomidate‐induced myoclonus [9].

In summary, these findings suggest that pretreatment with low‐dose midazolam, low‐dose etomidate, or TAES may reduce etomidate‐related adverse effects, particularly injection pain, without compromising hemodynamic stability. However, the observed reductions in myoclonus should be interpreted with caution, as the overall between‐group difference did not reach statistical significance. The three interventions may operate through distinct mechanisms, including GABAergic modulation with midazolam, receptor desensitization with low‐dose etomidate, and activation of endogenous opioid pathways with TAES.

However, interpretation of treatment effects should consider baseline imbalances observed after randomization, which may introduce residual confounding despite adjusted analyses.

Outcome assessments were performed before administration of opioids and neuromuscular blocking agents, reducing the likelihood that these medications influenced observed motor responses. Nevertheless, pharmacologic interactions during peri‐induction cannot be completely excluded in clinical settings.

In clinical practice, selection of a preventive strategy should account for individual patient characteristics, drug availability, potential adverse effects, and the feasibility of nonpharmacological alternatives. As a non‐invasive intervention, TAES may represent a viable option when pharmacologic premedication is contraindicated or not preferred.

Larger multicenter randomized controlled trials are needed to validate these findings and further assess pharmacological and nonpharmacological strategies, individually or in combination, for preventing etomidate‐induced myoclonus and injection pain.

## 5. Limitations

This study should be interpreted in light of several limitations. First, the trial was conducted in a single center with a modest sample size, limiting statistical power and external validity.

Second, despite randomization, clinically relevant baseline imbalances were observed (notably sex distribution and comorbidity profiles), which may have introduced residual confounding; adjusted analyses were therefore conducted as sensitivity assessments.

Third, the absence of a sham TAES comparator limits the causal interpretation of TAES‐specific effects and underscores the need for future sham‐controlled trials.

## 6. Conclusion

In this single‐blind, randomized clinical trial, premedication with low‐dose midazolam, low‐dose etomidate, or TAES completely prevented observable etomidate injection pain and reduced the incidence of etomidate‐induced myoclonic movements by a clinically meaningful amount compared with control, without evidence of hemodynamic instability or major adverse events. The reduction in myoclonus did not reach statistical significance, suggesting these findings should be interpreted as a favorable trend rather than definitive proof of efficacy.

Consistent with these findings, all three interventions were also associated with a reduction in the composite primary outcome (defined as injection pain and/or myoclonus); however, this effect was primarily driven by the prevention of injection pain and did not uniformly reach statistical significance across all comparisons. Therefore, the observed benefit should be interpreted cautiously.

Given their safety profiles and practicality, low‐dose midazolam, low‐dose etomidate, and TAES may be considered options to mitigate the common adverse effects of etomidate in routine clinical practice. In light of previous work showing that opioid‐based pretreatments such as sufentanil can be even more effective than midazolam in preventing myoclonus, further research comparing a broader range of pharmacologic and nonpharmacologic strategies is warranted to identify the most effective and safest approach for individual patients.

## Author Contributions

Mehran Rezvani Habibiabadi, Behzad Nazemroaya, and Reza Tanhaei contributed to the conception and design of the study, patient recruitment, data collection, and data interpretation. Behzad Nazemroaya performed the statistical analysis. Mehran Rezvani Habibiabadi, Behzad Nazemroaya, and Reza Tanhaei participated in drafting and critically revising the manuscript.

## Funding

This research did not receive any specific grant from funding agencies in the public, commercial, or not‐for‐profit sectors.

## Disclosure

All authors approved the final version for submission.

## Ethics Statement

This study was approved by the Ethics Committee for Research at Isfahan University of Medical Sciences, Isfahan, Iran (approval number IR.MUI.MED.REC.1404.070).

## Consent

Written informed consent was obtained from all participants prior to enrollment in the study.

## Conflicts of Interest

The authors declare no conflicts of interest.

## Data Availability

The datasets generated and/or analyzed during the current study are not publicly available due to ethical and privacy restrictions (i.e., they contain sensitive clinical information with the potential to re‐identify individuals). Deidentified data may be made available from the corresponding author upon reasonable request, subject to approval by the relevant ethics committee/institution and, where applicable, execution of a data use agreement.
